# Expansion of medical vocabularies using distributional semantics on Japanese patient blogs

**DOI:** 10.1186/s13326-016-0093-x

**Published:** 2016-09-26

**Authors:** Magnus Ahltorp, Maria Skeppstedt, Shiho Kitajima, Aron Henriksson, Rafal Rzepka, Kenji Araki

**Affiliations:** 1 Stockholm, Sweden; 2 Department of Computer Science, Linnaeus University/Gavagai, Växjö/Stockholm, Sweden; 3 Graduate School of Information Science and Technology, Hokkaido University, Sapporo, Japan; 4 Department of Computer and Systems Sciences (DSV), Stockholm University, Stockholm, Sweden

**Keywords:** Japanese language processing, Medical vocabulary expansion, Distributional semantics, Random indexing, Agglomerative hierarchical clustering

## Abstract

**Background:**

Research on medical vocabulary expansion from large corpora has primarily been conducted using text written in English or similar languages, due to a limited availability of large biomedical corpora in most languages. Medical vocabularies are, however, essential also for text mining from corpora written in other languages than English and belonging to a variety of medical genres. The aim of this study was therefore to evaluate medical vocabulary expansion using a corpus very different from those previously used, in terms of grammar and orthographics, as well as in terms of text genre. This was carried out by applying a method based on distributional semantics to the task of extracting medical vocabulary terms from a large corpus of Japanese patient blogs.

**Methods:**

Distributional properties of terms were modelled with random indexing, followed by agglomerative hierarchical clustering of 3 ×100 seed terms from existing vocabularies, belonging to three semantic categories: Medical Finding, Pharmaceutical Drug and Body Part. By automatically extracting unknown terms close to the centroids of the created clusters, candidates for new terms to include in the vocabulary were suggested. The method was evaluated for its ability to retrieve the remaining *n* terms in existing medical vocabularies.

**Results:**

Removing case particles and using a context window size of 1+1 was a successful strategy for Medical Finding and Pharmaceutical Drug, while retaining case particles and using a window size of 8+8 was better for Body Part. For a 10*n* long candidate list, the use of different cluster sizes affected the result for Pharmaceutical Drug, while the effect was only marginal for the other two categories. For a list of top *n* candidates for Body Part, however, clusters with a size of up to two terms were slightly more useful than larger clusters. For Pharmaceutical Drug, the best settings resulted in a recall of 25 % for a candidate list of top *n* terms and a recall of 68 % for top 10*n*. For a candidate list of top 10*n* candidates, the second best results were obtained for Medical Finding: a recall of 58 %, compared to 46 % for Body Part. Only taking the top *n* candidates into account, however, resulted in a recall of 23 % for Body Part, compared to 16 % for Medical Finding.

**Conclusions:**

Different settings for corpus pre-processing, window sizes and cluster sizes were suitable for different semantic categories and for different lengths of candidate lists, showing the need to adapt parameters, not only to the language and text genre used, but also to the semantic category for which the vocabulary is to be expanded. The results show, however, that the investigated choices for pre-processing and parameter settings were successful, and that a Japanese blog corpus, which in many ways differs from those used in previous studies, can be a useful resource for medical vocabulary expansion.

## Introduction

The ability to recognise named entities in text, and then to map these to concepts in medical ontologies, is key for most systems that rely on medical language processing, such as information extraction systems for syndromic surveillance [1], automatic detection of adverse drug events [2–4] and co-morbidity analyses [5]. As a result, much research has been conducted, and many systems developed, with the aim of improving these building blocks of medical information processing systems.

There are a number of systems for performing mapping to specific vocabulary concepts, for instance MetaMap [6], IndexFinder [7], MedLEE [8] and SAPHIRE [9] — all of these map entities to concepts in the Unified Medical Language System (UMLS) [10]. Although mapping systems typically employ techniques for handling abbreviations, misspellings, inflections and word order differences [11, 12], the availability of a comprehensive vocabulary is perhaps the most important prerequisite for performing high-quality concept mapping. Extensive vocabularies are also essential for named entity recognition approaches that rely on vocabulary mapping [13, 14] as well as for medical text simplification [15]. Vocabularies have, moreover, also been shown to be useful for generating features to be used when training machine learning models to recognise named entities [16].

There are a number of extensive medical vocabularies, many of which are included in the UMLS Metathesaurus [10]. These vocabularies are, however, not available in all languages; in languages for which they are available, they are often less extensive than resources for English. Although also less extensive vocabularies have been shown useful for medical text mining [17], limitations in the vocabularies used can lead to decreased performance. Previous studies on applying Swedish UMLS resources (which are less extensive than resources for English) for detecting entities in Swedish clinical text showed, for instance, a recall of 55 % for detecting Disorders, 33 % for detecting Findings, 80 % for detecting Body Parts [18], and a recall of 74 % for detecting Pharmaceutical Drugs [19]. Controlled medical vocabularies are, moreover, typically focused on terms from the professional medical language, despite the important applications of text mining from social media, such as syndromic surveillance [20, 21] or detection of adverse drug reactions [22]. The fact that terms used by laymen to describe medical concepts often differ from those used by health professionals [15, 23] render the controlled medical vocabularies less useful for text mining from social media [22, 24] as well as for applications such as medical text simplification [25].

To improve the performance of concept mapping, entity recognition and medical text simplification, existing vocabularies thus need to be expanded and adapted to the text domain in which such systems are to be employed. To manually expand and adapt vocabularies to variations in language between different text genres and over time is, however, expensive and time-consuming. Methods that can support this process are therefore valuable, for instance methods for semi-automatic vocabulary expansion.

Many previously explored methods for medical vocabulary expansion [26–28] rely on terms and abbreviations being explicitly defined or classified in the text. There are, however, many medical text genres in which the terms used are only very rarely explained to the reader, e.g., the narrative text of health records and the above mentioned social media texts. For such genres, other strategies for vocabulary expansion are required. One strategy is to use existing vocabularies as a starting point, and search for additional terms that occur in similar contexts as the existing vocabulary terms. This can be motivated by the distributional hypothesis, which states that words which occur in similar contexts often have similar meanings [29].

For most languages, there is a limited availability of large medical/biomedical corpora that can be used for research. As a result, research on medical vocabulary expansion, using distributional semantics methods developed for large corpora, e.g., random indexing or word2vec, has typically focused on English vocabularies [30–34], and on vocabularies for relatively similar languages, e.g., Swedish [35–37]. Less work has been carried out on vocabulary expansion using languages not related to English, or on medical text genres with laymen authors.

Outside of the medical domain, the variability of studied languages is larger, and distributional semantics has been applied to e.g., Japanese text, in studies using context in the form of noun-verb and noun-noun dependencies [38, 39]. Research on the adaption of distributional semantics to Japanese corpora, using context information in the form of several neighbouring words, has, however, not always been successful [40]. This indicates that, although distributional semantics methods are often claimed to be language independent, there might be languages for which adaptions of standard configurations and pre-processing methods are required. One difficulty associated with Japanese emerges from the fact that white space is normally not used ([41], p. 17). The standard approach for segmentation used in distributional semantics, in which the white-space segmented word forms the basic semantic unit (with special handling of punctuation marks and sometimes also of abbreviations), can therefore not be employed. Grammatical differences between languages could pose another challenge and might entail that the pre-processing choices that are most optimal for, e.g., English are not necessarily suitable for, e.g., Japanese.

In this study, we aim to investigate the possibility of expanding medical vocabularies by applying distributional semantics on a medical corpus that differs from corpora used in previous studies. We use a corpus that differs in terms of language as well as text genre, namely a corpus of Japanese patient blog texts. We focus on three semantic categories that are highly relevant for — just to name a few — the aforementioned tasks of syndromic surveillance and detection of adverse drug events: the semantic categories *Medical Finding*, *Pharmaceutical Drug* and *Body Part*. We will compare different pre-processing strategies and parameter settings with the aim of investigating whether standard pre-processing and parameter settings need to be adapted to (1) the Japanese language, (2) the text genre, or (3) to the semantic category.

## Background

There are a number of methods designed to support the creation or expansion of vocabularies that, more specifically, categorise words into semantic categories [42] or identify hyponyms of selected terms [43]. With the existence of semantic categories, (semi-)automatic vocabulary expansion can be seen as a word classification task, in which it should be determined whether an unknown word belongs to a certain semantic category or not. When categories have not been defined, however, word clustering can instead be considered as a means to discover potential semantic categories [44]. As there is extensive work on the definition of semantic categories within the medical domain, e.g., within UMLS [10], the first approach is typically taken for medical vocabulary expansion, i.e., classification of unknown words into pre-defined medical categories.

The material for creating or expanding a vocabulary could be an existing vocabulary, for instance when identifying synonym candidates by searching for similar term descriptions in a lexicon [45], or when translating a vocabulary from one language into another [46]. An alternative to using information from existing vocabularies is to use corpora for extracting term candidates for inclusion in a vocabulary. A frequently used approach is to find text patterns in which terms used are explained to the reader. From the text pattern “*term1* also known as *term2*” [47], for instance, it could be deduced that *term1* and *term2* are synonyms, while the text pattern “*term1* such as *term2*” could be used for extracting terms of a given semantic category [42]. These patterns can be either manually crafted or automatically extracted from the corpus. A related approach is to, instead of using explicit language patterns, rely on frequent co-occurrences in a large corpus between, e.g., terms and their hypernyms [48]. Another type of pattern-based vocabulary extraction from corpora is the construction of patterns for terms consisting of words with certain syntactic or semantic relations, e.g., automatically constructed patterns for extracting specific types of noun-verb pairs [49]. These corpora-based methods have been applied in the biomedical domain for synonym extraction [27, 47, 50] and for extracting terms of a certain semantic category [28, 48]. In the latter study, terms belonging to the semantic category Disease were automatically extracted by first extracting all sentences containing the word “disease” and thereafter using a known set of disease terms to train an SVM classifier to detect terms belonging to this semantic category. A precision of 38 % and a recall of 45 % were achieved when applying the automatically constructed vocabulary for vocabulary-based named entity recognition of diseases.

As mentioned in the introduction, it is also possible to extract terms on the basis of the contexts in which they typically occur [44]. This approach is more suitable to some types of corpora, e.g., blog posts or health record narratives, since it does not depend on hyper/hyponomy being explicitly stated in the text. Instead, it exploits the fact that words that frequently occur in similar contexts are likely to have similar meanings. Representation models for such term co-occurrence can, for instance, be probabilistic, such as in Brown clustering [51], or spatial models [52], in which term co-occurrences are given a geometric representation in the form of a vector space. Here, semantic similarity is based on geometric proximity. Zhang and Elhadad [32] have explored the approach of using distributional semantics for vocabulary expansion in the biomedical domain [32]. They constructed signature vectors for noun phrase chunks in a corpus based on the words included in the chunks, the surrounding context words (two words to the left and right, respectively, of the noun phrase chunk), as well on their inverse document frequency. As seed words, they used the terms of the relevant semantic categories available in UMLS. Cosine similarity was then measured between the noun phrase chunks in the corpus and the average of the seed terms’ signature vectors. All noun phrase chunks with a similarity to the average signature vectors above a certain threshold were considered as candidates for new terms belonging to the investigated semantic categories. For detecting named entities of the categories Medical Problem, precision scores of between 27 % and 28 % and recall scores of between 31 % and 34 % were achieved when applying their method on the three different clinical sub-corpora within the 2010 i2b2/VA challenge.

Most research on the expansion of medical vocabularies using distributional semantics in large corpora has been performed on English, but similar research using smaller corpora has been carried out on a larger variety of languages. From a French 85000 token coronary diseases corpus, for instance, unknown tokens with dependency relationships similar to known medical vocabulary terms have been automatically extracted [53, 54]. The same corpus has been used for evaluating techniques for building word space models specifically adapted to small corpora by generalising and normalising distributional contexts [55]. The techniques were evaluated for the extent to which the neighbours in the semantic word space had a semantic relation in existing medical resources, e.g., relations of synonymy and co-hyponymy.

Random indexing is another spatial model of distributional semantics that can be used to build a semantic space. This method was originally proposed by Kanerva et al. [56] to deal with the performance problems, in terms of computational cost, that were associated with the commonly used models of distributional semantics at the time (latent semantic analysis/indexing). Due to its computational efficiency, random indexing remains a popular method when building distributional semantic models from very large corpora, e.g., large web corpora [57] or Medline abstracts [58]. There are, however, a number of other methods available, e.g., word2vec [59] and GloVe [60] that are also often used for creating spatial distributional semantics models from large corpora.

The efficiency of random indexing is achieved by circumventing the need to perform dimensionality reduction on the original term-by-*context* matrix (where the context can be defined as, e.g., a document or the surrounding words), which is an important component of many other models of distributional semantics [57]. Instead, a semantic space with a smaller, predefined dimensionality is created from the beginning. This is achieved by first assigning to each context feature (e.g., each unique term), a sparse, random vector of the required dimensionality. These vectors, called *index vectors*, are generated by randomly distributing a small set of non-zero (+1 and −1) values, with the remaining elements set to 0. If the dimensionality is sufficiently large in relation to the number of context features, the index vectors will, with a high probability, be nearly orthogonal to each other. The index vectors are only used for building the semantic space, which, instead, is composed of *semantic vectors*. The semantic vector of a term, of the same dimensionality as the index vectors, is obtained by adding up all the index vectors of the terms with which it co-occurs in a predefined context — typically a symmetric window of surrounding words. The similarity between terms can, e.g., be expressed by the cosine similarity between their vectors, i.e., the cosine of the angle (*θ*) between the semantic vectors **u** and **v**. This is computed as follows ([61], pp. 127–134):

$$\cos(\theta) = {\mathbf{u} \cdot \mathbf{v} \over \|\mathbf{u}\| \|\mathbf{v}\|} = \frac{\sum\limits_{i=1}^{n}{u_{i} \cdot v_{i}} }{ \sqrt{\sum\limits_{i=1}^{n}{(u_{i})^{2}}} \cdot \sqrt{\sum\limits_{i=1}^{n}{(v_{i})^{2}}} } $$

With random indexing and a window-based context definition, it is possible to create different types of semantic vectors. If the index vectors of the surrounding words are added to the target term’s semantic vector as-is, the resulting semantic vectors are known as *context vectors*. This, however, entirely ignores word order within the context window. There is also a version of random indexing, sometimes called random permutation, in which this is taken into account. It does so by rotating the elements in the index vector one step to the left or right, depending on if the context term appears to the left or right of the target term. When the permuting of index vectors is performed in this manner, the semantic vectors are denoted *direction vectors*. Random permutation spaces with direction vectors have been shown to better detect synonymy than random indexing spaces with context vectors [62].

There is a previous study, in which a random indexing space constructed from Swedish medical journal text was used for expanding a Swedish medical vocabulary list, consisting of MeSH terms denoting Medical Finding and Pharmaceutical Drug [36]. Using a set of 91 seed terms for each of the two semantic categories, it was possible to extract 53 % of the 90 expected Medical Findings and 88 % of the 90 expected Pharmaceutical Drugs among the top 1000 retrieved terms. The manual evaluation of precision showed results of 80 % for top 50 and 68 % for top 100 for Medical Finding and 64 % for top 50 and 47 % for top 100 for Pharmaceutical Drug. In that study, as well as in the previously mentioned study in which distributional semantics was used for expanding medical vocabularies [32], terms belonging to semantic categories given by currently available vocabularies (e.g., the categories Medical Finding and the Pharamceutical Drug) were treated as belonging to one distributionally similar category of terms. In both studies, the criterion for ranking unknown words as potential candidates was, therefore, based on similarity to all of (or to the average of) the seed terms from one of the categories in the existing vocabularies. This is not, however, necessarily a good strategy since there might be a number of distributional sub-clusters within each semantic category of the existing vocabularies. If such sub-clusters are positioned at large distances from each other in the semantic space, this might have the effect that words that are not part of these sub-clusters, but close to two or more clusters, will incorrectly receive a higher ranking than words that are close to the centroids of the sub-clusters. This was shown to be the case in a study using distributional semantics for expanding a Swedish vocabulary of cue terms for uncertainty and negation [63]. The strategy of first clustering the seed terms used into more distributionally similar subsets and thereafter using similarity to the centroids of these subsets as the criterion for ranking unknown words outperformed the strategy of treating the seed terms used as one single distributionally similar category of terms.

We will take the possibility into account that this might be the case also for the three semantic categories that we investigate here, i.e., Medical Finding, Pharmaceutical Drug and Body Part, and therefore study the effect of dividing seed terms of these categories into smaller, more distributionally similar, subsets before using them for vocabulary expansion.

## Materials

Two types of materials were used: a blog corpus and existing Japanese vocabularies.

### Corpus

The corpus used is a Japanese blog corpus from the TOBYO site, which collects blogs written by patients and/or their relatives [17]. After a first normalising step described below, the corpus contained 270 million characters and after pre-processing, also described below, it contained 50 million semantic units (2.5 million unique).

### Vocabularies

Since the semantic spaces were evaluated for their ability to expand vocabularies with terms belonging to the three semantic categories *Medical Finding*, *Pharmaceutical Drug* and *Body Part*, Japanese terms belonging to these categories were gathered from existing vocabularies. These were then used both as seed terms and as evaluation data.

For Medical Finding, the following terms were used: MeSH terms classified under the nodes *Diseases (C)* and *Mental disorders (F03)* [64], all MedDRA/J terms except those classified as *investigations*, *social circumstances* and *surgical and medical procedures* [65, 66], as well as terms from the Byomei diagnosis list [67]. For Pharmaceutical Drug, the following terms were used: MeSH terms classified under the node *Chemicals and Drugs (D)* and pharmaceutical brand names available at the TOBYO site [68]. For Body Parts, the following terms were used: MeSH terms under the node *Anatomy (A)* except those under the sub-nodes *Plant Structures (A18)*, *Fungal Structures (A19)*, *Bacterial Structures (A20)* and *Viral Structures (A21)*, as well as terms from a language education web page listing body parts in Japanese [69]. The Japanese translations of MeSH and MedDRA were obtained from the U.S. National Library of Medicine.^1^

Vocabulary terms occurring more than 50 times in the segmented corpus as a semantic unit in the context of a sentence were included in the set of terms used. Terms in existing vocabularies that were segmented into several semantic units were therefore excluded, as were infrequent terms, due to the weak statistical foundation for their context vectors. The number of terms in each semantic category, before and after the frequency filtering, is shown in Table 1.

**Table 1:** Vocabulary size

	Medical finding	Pharmaceutical drug	Body part
	(*#* semantic	(*#* semantic	(*#* semantic
	units)	units)	units)
All terms in used vocabularies	77350	27912	2960
More than 50 occurrences in the segmented corpus as a semantic unit in the context of at least one other semantic unit	753	276	214

## Methods

In addition to not being able to employ the standard segmentation approach of white space tokenisation, we identified a number of grammatical differences between Japanese and languages similar to English that might be relevant when constructing distributional semantics models. A morphologic normalisation in the form of a total lemmatisation is sometimes performed on corpora used for distributional semantics. Japanese is, however, highly agglutinative ([70], p. 297), with the possibility to add several suffixes to verbs and to one of the two Japanese adjective types, the verbal adjectives ([41], p. 45). The suffixes are, for instance, used for expressing negation ([41], p. 54), desire ([41], p. 111) or level of politeness/formality ([41], pp. 81–83). Full lemmatisation could therefore result in severe loss of information. In addition, distributional semantics studies on Germanic languages have shown that employing a small context window of co-occurring words (typically 1–2 preceding and following words) is most suitable when building models for word similarity [62]. This is not necessarily the case for languages with another sentence structure, such as Japanese. The basic word order of Japanese (SOV) is different from the word order of English, and it is also relatively free since the function of a word (e.g. whether it is a topic, subject or object) is indicated by case particles ([41], pp. 35-38). Therefore, another context window size might be more appropriate for Japanese. Also the stop word filtering, often used for e.g., English vocabulary extraction [62], might have to be adapted to Japanese, possibly retaining the frequently occurring case particles.

### Previously performed experiments

We have previously performed preliminary experiments using random indexing for extracting Medical Findings, Pharmaceutical Drugs and Body Parts from a Japanese blog corpus [71]. In these experiments, we compared three different corpus pre-processing versions to investigate the identified grammatical differences. In the first pre-processing version, the corpus was fully lemmatised and stop word filtering was performed by removing all semantic units except verbs, adjectives and nouns/pronouns. In the second version, parts of the information contained in the suffixes, which potentially has a large impact on the semantics of the surrounding semantic units, were retained. This included polarity (negation or affirmation), grammatical mood and voice, while e.g., formality level and tense were excluded. In the third version, case particles were also retained to study if this could compensate for the relatively free word order of Japanese. We also experimented with different context window sizes, constructing distributional semantic spaces with four different window sizes for each pre-processing version, using a context window of 1+1, 2+2, 4+4 and 8+8 surrounding semantic units.

The results from these initial experiments showed that the optimal window size and pre-processing technique was dependent on which semantic category was targeted. For extracting Medical Findings and Pharmaceutical Drugs, the two versions in which case particles were removed outperformed the version in which they were retained, while a context window size of 1+1 was optimal. For Body Parts, on the other hand, better results were obtained when case particles were retained. Variation in context window size had hardly any effect on the results for this semantic category when case particles were retained, but marginally better recall was obtained with a window size of 8+8.

### Aim of the performed experiments

The previously performed experiments revealed substantial differences in the optimal pre-processing choices for the investigated semantic categories: removing case particles and using the smallest context window was the most suitable for Medical Finding and Pharmaceutical Drug, whereas retaining case particles and using the largest context window was the most suitable for Body Part. In the previous study, however, a very simple method for leveraging the position of the seed terms in the semantic space was used [36], in which a *summed similarity* to a semantic category for every term in the random indexing space was calculated. The calculation was carried out by summing the cosine of the angle ($\theta _{\bar {u},\bar {s}}$) between the context vector of the semantic unit $(\bar {u})$ and the context vector of each term $(\bar {s})$ in the set of seed terms (*S*) of the semantic category in question.

$$ {summed similarity}(\bar{u}) = \sum_{\bar{s} \mathop \in S} \cos(\theta_{\bar{u},\bar{s}}) $$

It was thus not taken into consideration that each one of the three investigated semantic categories might in reality consist of a number of smaller distributionally similar sub-clusters, and that a more successful strategy for expanding a vocabulary might be to use proximity to these sub-clusters rather than proximity to all seed terms.

In addition, in the previously performed experiments, only a small vocabulary resource was used for evaluating the large semantic category Medical Finding, and the evaluation was only performed for two randomly selected sets of seed terms, which might make the results difficult to generalise for seed terms of the three investigated categories in general.

Given the results and the limitations of the preliminary experiment, two hypotheses were posed:

- Dividing the seed terms into sub-clusters and using similarity to their centroids as the ranking criterion is a more successful strategy than using the method of *summed similarity* to all seed terms.
- The appropriate choice of corpus pre-processing techniques and context window size depends on the semantic category of interest.

### Performed experiments

To investigate the posed hypotheses, an experiment was carried out in the following four steps: 1) Pre-processing of the corpus for random indexing; 2) construction of a semantic space using random indexing; 3) hierarchical clustering of vectors in the semantic space that correspond to seed words and production of ranked lists of terms according to their proximity to centroids of the constructed clusters (one list per maximum cluster size); 4) automatic evaluation of recall of the terms in the produced lists against a reference standard.

#### Pre-processing of the corpus for random indexing

Japanese is written using three sets of characters: *kanji*, the logographic characters borrowed from Chinese writing, are used for lexical morphemes; *hiragana*, one of the two syllabic character sets, are used for grammatical morphemes, both grammatical morphemes as individual words and as inflections; and *katakana*, the other syllabic character set, is used for loan words of non-Chinese origin ([72], pp. 184–192). There are, however, also some lexical morphemes for which there is no kanji and that, therefore, are written using hiragana, as well as morphemes that are commonly written using hiragana, despite a possibility to use a kanji character. The use of logographic characters often makes the morphological boundaries in compounds more evident than what is the case in a phonetic writing system. This has been used in previous studies, in which the morphological compounds of medical terms in languages with phonetic writing systems have been determined by mapping them to their corresponding Japanese term [73]. In contrast to many other writing systems, however, word boundaries are not marked by white space in Japanese ([72], pp. 184–192). This has the effect that white space tokenisation cannot be applied, which is a standard tokenisation method used in many previous distributional semantics studies.

The absence of white space was addressed by applying a number of steps for creating a segmented version of the corpus. A basic pre-processing was first carried out by removing smileys and sentences solely containing Latin characters, as well as normalising the hiragana and katakana characters by transforming half-width forms into the corresponding full-width form. Thereafter, the segmented version of the corpus was built in the following two steps: (a) applying the dependency parser CaboCha to the corpus [74] and (b) applying the semantic role labeller ASA [75] to the parsed corpus.

Two different versions of the tokenised corpus were then created to investigate the two different pre-processing strategies that had been the most successful in the previously performed experiments. In the first version, only semantic units classified by CaboCha as either a verb (not including helper verbs or copula), an adjective (including verbal adjectives, adjectival nouns and adverbial derivations of adjectives) or a noun (including pronouns) were retained; all other semantic units were removed. In addition, all verbs and verbal adjectives were fully lemmatised (as nouns, pronouns and adjectival nouns are not inflected in Japanese, they cannot be lemmatised). In the second version of the corpus, case particles were retained, in addition to verbs, adjectives and nouns/pronouns. Verb and verbal adjective inflections indicating polarity (negation/affirmation), grammatical mood (subjunctive/imperative/optative/interrogative/indicative) and voice (passive/causative/potential/active) were also retained in this version of the corpus.

Apart from constructing semantic units based on the constituent and morpheme boundary information given by CaboCha, ASA also provides the inflection type information used in the pre-processing. The output of semantic role labels, which is also given by ASA was, however, not used.

#### Construction of a semantic space using random indexing

For the corpus version in which case particles were removed, a random indexing space with a window size of 1+1 was created, and for the version with retained case particles, a random indexing space with a window size of 8+8 was created. The choices were based on the window sizes that were the most successful for the different pre-processing techniques in the previously performed experiments [71].

The parameter settings of the constructed spaces were generally based on standard settings for random indexing [62], i.e., using random permutation with 2000 dimensional direction vectors, with 10 non-zero elements in the index vectors. All semantic units in the context windows used were given equal weight.

In initial tests using the blog corpus for constructing random indexing spaces, index vectors corresponding to sentence boundaries were added to the semantic vector of a semantic unit whenever it occurred near the beginning or end of a sentence – a setting which has previously been used for, e.g., Swedish medical journal text [36]. This, however, led to semantic spaces in which a majority of the context vectors were positioned within close proximity to each other in the constructed space, probably since blog texts to a larger extent than, e.g., medical scientific text, consist of sentences with very few semantic units. These index vectors indicating sentence beginning and sentence ending were therefore removed, leading to a better distribution of the context vectors.

#### Clustering of seed terms

The general approach for generating a list of candidate terms for possible inclusion in the vocabulary was to use a set of seed terms for ranking all words in the corpus not in the seed term set (the unknown terms) according to their proximity to the seed terms in the semantic space. For each one of the three investigated semantic categories, the following was carried out.

Agglomerative, hierarchical clustering ([76], p. 700) was applied to the direction vectors of the seed terms. This was carried out by first assigning each vector its own cluster. The pairwise distances between clusters were thereafter calculated, and the two clusters with the largest cosine similarity between their respective centroids were then merged into a new cluster. This hierarchical clustering process was iteratively repeated until all the vectors of all seed terms of the semantic category formed a single cluster.

The cluster sizes that were most appropriate for the task were determined by successively moving upwards in the tree of the created clusters. First, clusters consisting of a maximum of one term were used for creating the ranked list of unknown terms (i.e., the case in which each seed term was treated as its own cluster). Then, clusters consisting of a maximum of two terms were used and the maximum cluster size was then successively increased until the root of the tree was reached. In each step in this process, a ranked list of candidate terms was created by ordering the unknown terms according to cosine similarly to their most closely located cluster centroid. That is, a similarity score was computed for each unknown term in the corpus ($\bar {u}$) by calculating the cosine of the angle $(\theta _{\bar {u},\bar {c}})$ between the unknown term and each of the centroids ($\bar {c}$) in the set of constructed centroids (*C*) and returning the largest cosine similarity score. The unknown terms were subsequently ordered according to decreasing similarity score.

$$ {cluster similarity}(\bar{u}) = \underset{\bar{c} \mathop \in C}{\operatorname{max}}\cos(\theta_{\bar{u},\bar{c}}) $$

As a final step, in order to enable comparison to the method from the previously performed experiment, the *summed similarity* method was also used for ranking the unknown terms.

#### Evaluation

The existing vocabulary terms in each semantic category were divided into two sets: one set of 100 seed terms and one set of evaluation terms, comprising the remaining terms (*n* terms). A situation was thus simulated, in which there is 100 terms in an existing vocabulary for each semantic category, while the remaining *n* terms represent new terms that ought to be added to the vocabulary. Recall for retrieving the terms in the evaluation set was measured for the top *n*, 2*n*… 10*n* candidates in the constructed lists. This simulates e.g., a medical terminologist manually scanning the top *n*, 2*n*… 10*n* candidate terms in search of new terms to add to a medical vocabulary.

To make the results less dependent on which terms were selected for the set of seed terms and which were used in the reference standard, a bootstrap resampling [77] approach was taken, in which the experiment was repeated 500 times, each time with a new random selection of seed terms. The final results were obtained by averaging the recall values obtained for each resampling.

A very crude baseline was also calculated in order to give an idea of how well the method performs in comparison with a list of randomly extracted semantic units from the corpus. When listing all semantic units that occur more than 50 times in the corpus in a random order, on average every *x*th item in the list would be a semantic unit from the reference standard. The top *t* terms in the list would thus on average contain *t*∗(1/*x*) terms from the reference standard. There are a total of 43050 semantic units that occur more than 50 times in the corpus, and if *n* is the number of semantic units in the reference standard for a given category, then *x* is 43050/*n*. Thereby, *t*∗(1/*x*) is *t*∗(*n*/43050), and the top n semantic units in this list would therefore on average contain *n*∗(*n*/43050) of the semantic units in the reference standard, the top 2n terms 2*n*∗(*n*/43050), and so on.

In addition to the automatic calculation of recall against the reference standard, an analysis was performed of factors influencing whether a reference standard term was often or seldom included as a highly ranked candidate. A manual analysis was also carried out to get an understanding of what terms were suggested as highly-ranked candidates, apart from those included in the reference standard. This manual analysis was carried out for a list of unique terms from the top 100 candidates in each fold, when using the best settings for each of the three categories.

## Results

**Fig. 1:**
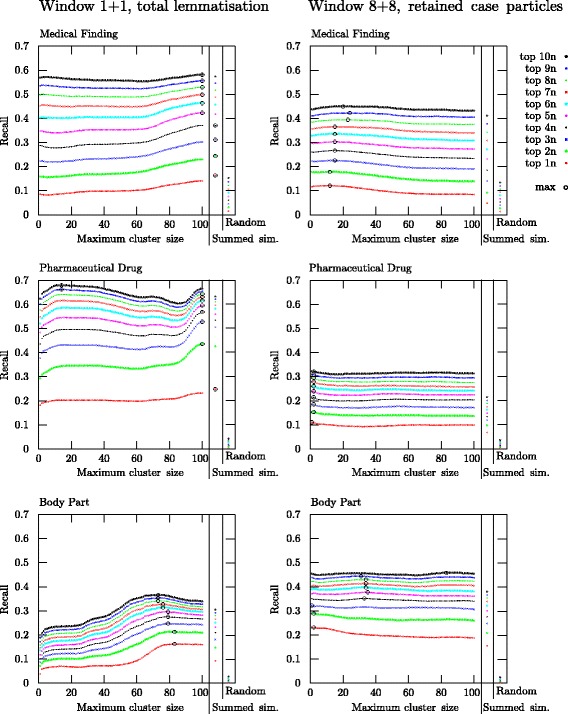
Recall for retrieving semantic units belonging to the three investigated semantic categories

The results, presented in Fig. 1, provide a good basis for accepting the second hypothesis, i.e., that a larger window size and retained case particles and inflections is a more suitable setting for retrieving term candidates for the semantic category Body Part, while a small window size and the removal of case particles and inflections is more suitable for the semantic categories Pharmaceutical Drug and Medical Finding. The best average recall values for a window size of 1+1 and a window size of 8+8 are also shown in Table 2 for top *n* candidates and in Table 3 for top 10*n* candidates. A 95 % confidence interval is given by the 2.5 %- and 97.5 %-percentiles of the 500 recall values obtained by bootstrap resampling [77].

**Table 2:** Best results for top n. For window sizes 1+1 and 8+8

% Window size 1+1	Best strategy	Average	2.5 % percentile	97.5 % percentile	Variance
Medical Finding	summed similarity	**16.3 %**	13.9 %	19.2 %	0.0002
Pharmaceutical Drug	summed similarity	**24.8 %**	20.1 %	29.4 %	0.0006
Body Part	cluster level 83	16.2 %	7.5 %	21.9 %	0.0011
Window size 8+8	Best strategy	Average	2.5 % percentile	97.5 % percentile	Variance
Medical Finding	cluster level 12	12.1 %	9.1 %	14.9 %	0.0002
Pharmaceutical Drug	cluster level 1	11.1 %	7.3 %	14.7 %	0.0004
Body Part	luster level 2	**23.1 %**	18.4 %	28.1 %	0.0006

The best results are shown in bold face

**Table 3:** Best results for top 10n. For window sizes 1+1 and 8+8

Window size 1+1	Best strategy	Average	2.5 % percentile	97.5 % percentile	Variance					
Medical Finding	cluster level 100	**58.1 %**	55.3 %	60.8 %	0.0002					
Pharmaceutical Drug	cluster level 14	**67.9 %**	56.2 %	77.4 %	0.0029					
Body Part	cluster level 73	36.6 %	20.6 %	46.5 %	0.0036					
Window size 8+8	Best strategy	Average	2.5 % percentile	97.5 % percentile	Variance					
Medical Finding	cluster level 20	44.9 %	40.5 %	49.7 %	0.0006					
Pharmaceutical Drug	cluster level 2	32.2 %	26.6 %	37.9 %	0.0010					
Body Part	cluster level 83	**45.7 %**	38.6 %	51.8 %	0.0011					

The best results are shown in bold face

The largest difference between the two settings was observed for the category Pharmaceutical Drug: the semantic space with a window size of 1+1 resulted in a maximum recall average of 25 % for top *n* and a maximum recall average of 68 % for top 10*n*, while the corresponding scores for the semantic space with a context window of 8+8 were 11 % and 32 %, respectively. Likewise, for Medical Finding, better results were achieved with the 1+1 space at each of the ten measurement points; however, the difference is smaller than for Pharmaceutical Drug, with maximum average recall values of 16 % for top *n* and 58 % for top 10*n* for the 1+1 space versus 12 % and 45 % for the 8+8 space. For Body Part, the reverse results were observed, i.e., that better results were achieved with the 8+8 space at each measurement point, with 23 % recall at top *n* and 46 % recall at top 10*n* versus 16 % recall and 37 % recall with the 1+1 space. At the top 10*n* level for window size 1+1 for Pharmaceutical Drug and especially for Body Part, the results had a larger variance than for the other results shown in Tables 2 and 3, indicating that the results for these categories and settings were more dependent on what terms were used as seed terms.

When comparing the results of the three categories, it can also be concluded that, for a candidate list of 10*n* candidates, the evaluated method is most successful for the category Pharmaceutical Drug, followed by Medical Finding, for which the maximum recall average is 10 percentage points lower, while it is yet another 13 percentage points lower for Body Part. For the top *n* candidates, on the other hand, the best results were achieved for Pharmaceutical Drug and Body Part, with slightly lower results for Medical Finding.

With respect to the first hypothesis, i.e., that a clustering approach would be more successful than the simple *summed similarity* method, the results are less evident, also when focusing on the context window size and pre-processing strategy that was the most successful for each category. To use the centroid of all seed terms was more successful for retrieving Body Part terms than to use the *summed similarity* method. There was, however, a very small difference between different cluster levels for this semantic category, except for the top *n* and 2*n* candidates, for which it was slightly more successful to use proximity to centroids of seed term clusters of size 2 as the ranking criterion. Likewise, for the category Medical Finding, there were no large differences between different cluster levels, but minimally better results were achieved when either using the centroid of all seed terms or the *summed similarity* method, depending on how many candidate terms were taken into account. The category Pharmaceutical Drug was the only category for which the results varied for different cluster levels for a longer candidate list. Using the centroids of clusters with a maximum size of 14 gave, on average, the best results with a candidate list of top 10*n* and 9*n*. No clear conclusions can, however, be drawn for this category either, since either the *summed similarity* method or using the centroid of all seed terms was more successful when measuring the recall for a shorter candidate list. Especially for top 2*n* and top 3*n*, using proximity to the centroid of all terms outperformed the use of proximity to clusters up to a size of 90 seed terms.

It could also be observed that the curves have different forms depending on the two explored window sizes/pre-processing choices. For the 8+8 space, all curves are very flat, while for the 1+1 space, the use of different cluster levels has a bigger effect on the results.

### Analysis of retrieved entities

**Fig. 2:**
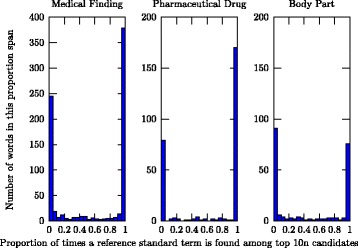
This illustrates how often a term is found when used as reference standard term. The first stack shows the number of terms that are correctly retrieved between 0 % and 5 % of the times they are used in the reference standard, the second stack shows the number of terms retrieved between 5 % and 10 % of the times, and so on. The statistics are shown for top 10*n* candidate terms (using cluster level 100 and fully lemmatised and stop word filtered corpus for Medical Finding and Pharmaceutical Drug and cluster level 34 with the corpus retaining more information for Body Part)

To investigate patterns for which terms were and were not retrieved by the evaluated methods, statistics of the proportion of times a term was retrieved when it appeared in the reference standard used were gathered. The best settings for each of the three studied categories were used, i.e., the setting that resulted in the best recall for a majority of the ten points of measurement. The results, visualised in Fig. 2, show that the distribution of retrieved terms among the top 10*n* candidate terms is highly skewed for all three investigated entity categories. Regardless of which set of seed terms is used, a large proportion of the terms are found in more than 95 % of the cases, while another large proportion is found in less than 5 % of the cases.

**Fig. 3:**
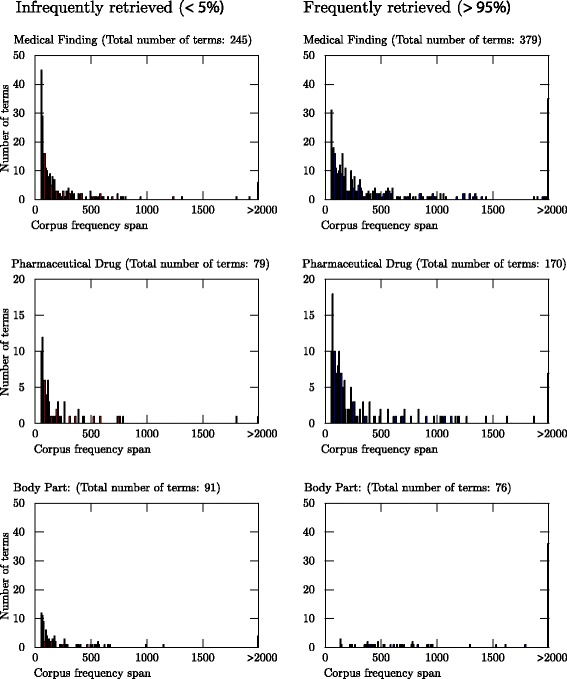
This illustrates the frequency of the terms in the TOBYO corpus for two opposite groups of terms used as evaluation data; those terms that were found in less than 5 % of the cases they were used as a reference standard term and those that were used in more than 95 % of the cases they were used as a reference standard term

For these two distinct groups of terms — those that were found in less than 5 % of the cases and those that were found in more than 95 % of the cases — the frequency of the terms in the TOBYO corpus was investigated and is visualised in Fig. 3. For the category Medical Finding, and even more clearly for Body Part, terms that occur relatively infrequently in the corpus are overrepresented among those that were retrieved in fewer than 5 % of the cases. For Body Part, these infrequently occurring terms were more typical for the specialised language of medical professionals than the more frequently occurring terms. Terms such as *tongue*, *waist/hips*, *finger* and *throat*, which are likely to occur naturally in laymen text, were often retrieved. Many more technical terms, such as *cranial nerve*, *pancreatic duct* and *nasal cavity*, were, on the other hand, never retrieved. For the category Medical Finding, however, no such evident difference between laymen language and professional language could be found between frequently and infrequently retrieved terms. The visualisation shows that increasing the frequency cut-off (which was 50 occurrences in the corpus) by another 10 or 20 occurrences would have resulted in a higher recall for the evaluation method applied, especially for Body Part, for which no infrequent terms were found.

For the category Pharmaceutical Drug (which contained terms under the MeSH node *Chemicals and Drugs*), there was also a trend of infrequently occurring terms being overrepresented among those retrieved in less than 5 % of the cases. The trend was, however, not as evident as for the other two categories. A brief inspection indicated that terms denoting chemicals often referred to in non-medical domains were more frequent among terms that were rarely found than among those that were often found. A manual classification of the terms used for evaluating the category Pharmaceutical Drug was therefore performed, in which the terms were classified according to the three groups: *a) Terms often used for denoting a typical pharmaceutical drug*, *b) Terms often used when referring to concepts not related to pharmaceutical drugs* and *c) Terms often used for pharmaceutical drugs as well as for concepts not related to pharmaceutical drugs*. The classification, which was performed by an annotator without knowledge of which terms were often or rarely retrieved, showed that among terms found in more than 95 % of the cases, 91 % belonged to the class *Terms often used for denoting a typical pharmaceutical drug*, while the same figure for terms found in less than 5 % of the cases was 34 %.

Examples of terms that were rarely found are *diamonds*, *vehicular emission* and *table salt*, while *cough suppressants*, *diclofenac* and *analgesic drugs* are examples of terms often found. As the patient blogs are not solely focused on medical issues, it is very likely that there are many terms under the MeSH node *Chemicals and Drugs* that occur frequently in the corpus, without referring to concepts related to pharmaceutical drugs.

### Analysis of highly ranked candidate terms not included in the reference standards

For producing lists of highly ranked terms not included in the reference standards used, the following was carried out for each one of the three semantic categories: The top 100 terms in the candidate list (produced using the best settings according to the criteria described above) were gathered from each one of the 500 folds, resulting in a list of 50000 items. The list was then reduced to include only one unique occurrence of each term (as most terms were on top 100 lists produced from many of the folds, most terms occurred several times in the gathered list). In addition, terms included in existing vocabularies were removed from the list.

This resulted in a list of 313 terms for Medical Finding (397 when including those found in existing vocabularies), 94 terms for Pharmaceutical Drug (143 including terms from vocabularies) and 407 terms for Body Part (485 including terms from vocabularies). The very large reduction of the lists when only keeping unique terms, shows the consistency of candidate terms generated between different folds. The lists of unique terms were then manually analysed by searching for categories of terms.

Around a fifth of the candidate terms for Medical Finding could be classified as belonging to this category. Among them were terms describing states of mind, most of them negative, e.g., *depression*, *anxiety/fear*, *lack of sleep* and  and *worry/pain*; compound terms, e.g., *blood vessel* + *pain* =*vascular pain* and *tear*+*eyes*=*teary eyes*; terms consisting of orthographic variants of those found in vocabularies, e.g., *breast cancer* and ; English and German loan words, e.g., *panic*, *trauma* and *complex*. Some of these might be possible to include in a medical vocabulary of more formal terms, while others would be typical to a resource of laymen terms. A type of expressions that might be considered too informal for the professional language are the double-form onomatopoetic words that were found among the new Medical Finding terms, e.g, *dizzy*, *completely exhausted/weak* and , *worn-out/shabby*. Despite their informal nature, they might, however, still be useful, for instance when mining for descriptions of patient reactions to drugs, and there are examples of such terms in the existing vocabularies used in the study, e.g., the term *feel sick/irritated*.

It is difficult to draw the exact line for when a described state of mind should be considered a Medical Finding; as a result, an additional 4 % of the candidate terms were not considered Medical Findings, but more general descriptions of state of mind, e.g., *loneliness*, *cowardice*, as well as hypernyms to terms describing states of mind, e.g., *feeling/mode* and *feeling/emotion*. Among the candidates not categorised as Medical Findings, there were also about the same amount of terms describing some kind of level or change of state, e.g., *change of mood*, *change* and , *half price* and *development*, of which some might be used for describing that there is a medically relevant change in the patient.

Around a tenth of the candidates described general phenomena, most of them phenomena that at least in some contexts could be described as negative, e.g., *bad habit/peculiarity*, *uproar/disturbance*, *trouble* and *problem/question*. Although some of these terms are likely to be semantically close to Medical Findings due to frequently occurring in similar negative contexts, there were also terms that typically occur in the context of descriptions of Medical Findings, e.g., the term *custom/habit*— that, for instance, is a component in the expression *life-style disease*— and the term *condition*, for instance used in an expression such as *be in a good state of health*. These kinds of terms might be too general to include in a vocabulary of professional language, but might be the expressions used by patients when describing their health and therefore an important resource for medical text mining from laymen texts.

The candidate terms for the category Pharmaceutical Drug could easily be divided into two main groups. The first group (53 %) were terms denoting pharmaceuticals; of which 28 % were trade names, e.g., *Tamiflu* and an abbreviated form of *Elental*; 50 % were specifications of types of pharmaceuticals, e.g., *painkiller*, *antipyretic*, *vitamin pills* and *sleeping pills* and ; and finally 22 % were orthographic and other versions of words for pharmaceuticals, e.g., honorific form , written with hiragana and katakana  and , a misspelling/pre-processing error , as well as versions of their physical forms, e.g., *pill* and *capsule*.

The second group of Pharmaceutical Drug candidate terms (46 %) were drinks, e.g., *coffee*, *milk* and *tea*, including three drink-related words *gulping/gulp down* and  and *straw*. Apart from the two groups pharmaceuticals and drinks, there was only one additional term, *endoscope*. The verb used in Japanese for taking a medicine is the same word used for drinking , which explains the large group of drinks in the candidate list.

The candidate terms for Body Part had a larger semantic diversity than those for Pharmaceutical Drug. Around 17 % of the terms denoted body parts/structures/fluids, e.g., *gums*, *cartilage*, *digestive organs* and *blood*. Of these terms 10 % were specifications of body parts, e.g., *right shoulder* and *left arm*; 23 % were orthographic variants or honorific forms, e.g., *head* written in katakana  and *stomach* written in hiragana .

The largest group (20 %) among the candidate terms for Body Part were, however, terms describing persons, many of them family members or persons associated with health care, e.g., *baby*, *twins*, *physician*, *patient* in polite form , *nurse* and *Japanese person*. Three other evident groups were Medical Findings (7 %), computer related terms (4 %) and animals (3 %), e.g., *wound*, , *infection*, *personal computer*, *monitor*, *fish* and *dog*. There were also terms semantically related to body parts, e.g., those denoting things that are physically close to or can be worn on the body, such as *pillow*, *wristwatch*, *trousers* or *smile/laughter*, as well as things that have a physical form similar to body parts, e.g., *container/vessel*, *balloon* and *pump*.

## Discussion

We have experimented with different pre-processing and parameter settings for expanding a vocabulary using distributional semantics on Japanese text, more specifically Japanese patient blog text and the three semantic categories Medical Finding, Pharmaceutical Drug and Body Part. As mentioned above, previous research on medical vocabulary expansion has mainly been conducted on English or similar languages, and also often on text genres in which the vocabulary used is sometimes defined or explained to the reader, which is not normally the case in the blog genre.

### Quality of the results compared to those of previous studies

Results obtained in previous studies are difficult to compare directly to those obtained here, as results are heavily dependent on, e.g., the evaluation strategies used. In previous approaches, in which automatically expanded vocabularies have been used for named entity recognition, the category Disease was recognised with a precision of 38 % and a recall of 45 % [28] and the category Medical Problem with precisions values between 27 % and 28 % and recall values between 31 % and 34 % [32]. Although these studies take a more indirect and application-oriented approach to evaluation than the one taken here, the results indicate that fully automatic generation of medical vocabularies is a difficult task, also for English, for which vocabulary expansion from corpora has been studied more thoroughly.

For the vocabulary extraction from the French coronary diseases corpus, *Diseases/Diagnoses* had a precision of 17 %, while *Chemicals, Drugs and Biological Products* had a precision of 38 % for the 24 and 8 new terms, respectively, that were assigned those semantic categories [54].

Some of the results obtained here for vocabulary expansion could therefore be described as more than acceptable; for instance, being able to expand a hypothetical seed vocabulary of 100 terms with at least 119 new Pharmaceutical Drugs by manually scanning a list of 1760 candidate terms (i.e., top 10*n* candidate), and at least 203 new Medical Findings by scanning through a list of 1959 candidates (i.e., top 3*n* candidates). Moreover, for the category Pharmaceutical Drug, scanning through the list of 1760 candidates resulted in 68 % of the terms in a current vocabulary being retrieved, which is a relatively high coverage for a relatively low-cost effort. For Medical Finding, a maximum recall of 58 % was achieved for the top 10*n* candidates. Although this would require the more laborious task of scanning through 6530 candidates, it could be motivated by the fact that the Medical Finding category contains many vocabulary terms (as can be seen when comparing the categories in Table 1). Scanning a list of 1140 (top 10*n*) candidates for the category Body Part resulted in at least 52 new Body Part terms being added to the vocabulary, which comprises 46 % of the expected terms, i.e., a lower result than for the other two categories. It should be noted that the number of new terms that would be retrieved at different lengths of the candidate list are described as “at least” *x* number of terms, since the candidate lists also contain instances of terms that are not yet included in available vocabularies, as was shown by the manual analysis of the top 100 candidate terms. In addition, the recall values would also be higher if a larger cut-off value for term frequency would be used, as low-frequency terms were overrepresented among terms not found. Therefore, the explored methods have an even larger potential than indicated by the recall scores.

The results previously obtained for extracting Medical Findings and Pharmaceutical Drugs from Swedish medical journal text [36] are slightly more comparable, as a seed term set of approximately the same size was used, and since the results were measured for a number of candidates close to 10*n*. When directly comparing the recall results, it can be concluded that higher recall was achieved for extracting Pharmaceutical Drugs from Swedish text (88 % compared to a top 10*n* recall of 68 % achieved here), while we here achieved a slightly higher recall for the category Medical Finding (58 % compared to the previously achieved recall of 53 %). Although, as previously mentioned, the results cannot be directly compared, they show that there are no dramatic differences stemming from the fact that we here expanded a vocabulary using distributional semantics on a Japanese blog corpus instead of using a corpus of a more formal text genre written in a Germanic language.

### Adaptions to Japanese text and to the blog genre

The quality of the results show that the general strategies for pre-processing the corpus were successful, i.e., using CaboCha and ASA, as well as removing all function words/all function words but case particles.

Vocabulary terms had to occur in the corpus as independent semantic units more than 50 times to be used as seed/evaluation terms, and it is likely that some of the many terms that were excluded from vocabulary lists used were excluded because they were multi-word terms and, thereby, had been segmented into multiple semantic units by CaboCha/ASA. This is, however, not specific to the use of CaboCha/ASA for creating semantic units, but is an even larger problem when creating semantic spaces built on corpora pre-processed with white-space tokenisation. Therefore, many of the vocabulary terms that were tokenised as independent semantic units by CaboCha/ASA, and also suggested as candidates for vocabulary expansion, would not have been suggested in the similar study conducted in Swedish [36], as they would have been divided into two different semantic units by the white-space tokeniser. (For instance *diabetes mellitus type 2*, Japanese: 2 , Swedish: *Typ 2-diabetes*). The difference is even larger between CaboCha/ASA tokenised Japanese and white-space tokenised English, as compound words are less frequent in English than in Swedish. Therefore, e.g., *skin diseases* (Japanese: , Swedish: *hudsjukdomar*) would be correctly tokenised for this purpose in Swedish and in CaboCha/ASA-tokenised Japanese, but not in English. This is typically dealt with by creating n-grams [30], or by compositional distributional semantics [78].

The removal of smileys and of the index vectors indicating sentence beginning and end were the only specific adaptions in the pre-processing and parameter settings that were performed for the blog text genre. Which vocabulary terms were used for the evaluation were, however, indirectly governed by the choice of text genre, as some of the terms in medical vocabularies are more frequent in the language used by patients. For the semantic category Body Part, terms occurring rarely in the corpus were very overrepresented among those that were seldom retrieved. These terms were also terms more typical for the language used by medical professionals than for the language used by patients, which functions as a reminder that vocabulary expansion from patient blogs can, and should, only aim at expanding medical vocabularies with terms that are included in the language used by patients.

### Differences between the three studied semantic categories

Apart from the more general conclusion that the pre-processing used was successful for Japanese text and for the genre of patient blogs, the most important conclusion that can be drawn from the conducted experiments is that different settings for expanding a vocabulary might be suitable for different semantic categories. The most suitable settings might also depend on the number of candidate terms that, e.g., a medical terminologist is willing to scan through. To construct sub-clusters of seed terms had, for instance, no (or very marginal) effect when extracting Medical Findings, or when using the best settings for extracting Body Parts from 10*n* candidate terms. When only looking at the *n* or 2*n* top candidates for Body Part, on the other hand, to use proximity to centroids of clusters with a maximum size of two seed terms in each cluster was most successful. To use proximity to centroids of clusters with a maximum size of 14 seed terms or to the centroid of all 100 seed terms, were successful ranking strategies when extracting Pharmaceutical Drugs from a term list of 10*n* candidates, while there was a dip in performance when using the centroid of clusters with a maximum size of 90 terms.

In addition, retaining case particles/inflections and using a large context window size was most successful when extracting Body Parts, while removing case particles/inflections and using a small context window was better for Medical Finding and Pharmaceutical Drug. The reason why different parameter settings are optimal for different semantic categories could be that Medical Finding and Pharmaceutical Drug are possible to distinguish from other semantic categories given neighbouring lexical words, such as the verb *take a medicine/drink* for pharmaceuticals, while adding additional information just adds extra noise. Terms from the category Body Parts, on the other hand, seem to occur in lexical contexts similar to those of terms belonging to other large semantic categories, e.g., terms describing persons and animals. Retaining more grammatical information, as well as using a larger context window, could, however, be a strategy that better distinguishes Body Parts from these other semantic categories.

In short, parameter settings need to be adapted to each semantic category for which the vocabulary is to be expanded. In a realistic setting, the aim would be to find the maximum number of new relevant terms of a semantic category given the currently available terms, rather than, as in this study, evaluate the suitability of a method for expanding a vocabulary, independently of which terms are already included in the vocabulary. All available vocabulary terms belonging to the semantic category of interest would in such a case be used as seed terms, and the most suitable parameter settings given this particular seed set should then instead be determined, e.g., by leave-one-out cross validation.

### Implications and future work

There are a number of English medical corpora in which entities of the three semantic categories explored in this study have been manually annotated [79–81]. In some of these studies, the laborious annotation effort has been facilitated by automatic pre-annotation built on the extensive medical vocabulary resources that are available for English [82]. For a language (or genre) with less extensive vocabulary resources, on the other hand, vocabulary-based pre-annotation might not be as useful. The results of our study show, however, that distributional semantics can be leveraged for semi-automatically extending an existing, small vocabulary. This might enable high-quality vocabulary-based pre-annotation also for a language with limited vocabulary resources. We believe that scanning through a list of candidate terms and determining which of these belong to a certain semantic category is faster than scanning text for these entities, especially since the results of our study show the potential for creating lists with a higher density of the relevant term candidates than would be the case when scanning through text. The results show that these methods, previously applied on English and languages similar to English, can also be successfully applied to text written in a very different language. Since the methods work for both language types, despite the existence of important orthographic and grammatical differences, we believe that there is a high potential for these methods to work well also on several other languages. We therefore hope that the implication of our study will be that medical annotation projects on corpora from languages with less extensive vocabulary resources will include an initial step in which medical terminologies are semi-automatically expanded by methods similar to those explored here. This would enable vocabulary-based pre-annotation also for such annotation projects.

Our future plans include the employment of this strategy for annotating the three investigated semantic categories in a subset of the TOBYO patient blog corpus. We aim to use all available vocabulary terms as seed terms, and determine what settings are most suitable for this larger seed term set by employing leave-one-out cross-validation. By applying distributional semantics on patient blogs, we aim to expand existing vocabularies with more terms that are typical to the language used by patients.^2^

To gather lists of terms belonging to certain semantic categories is sufficient for named entity recognition, but vocabulary lists are not enough to be able to perform concept mapping of detected entities. Future work, therefore, also includes strategies for positioning the gathered terms within the hierarchy of a vocabulary, either as a synonym to an existing vocabulary concept or as a new independent concept that is to be positioned as a hyponym to one of the existing vocabulary concepts. Distributional semantics could be applied also for this task, as has been shown in previous research [37].

Another future plan could be to automate the process of optimising the parameter settings for different semantic categories. Alternatively, it might be useful to explore if there are methods whose performance is more robust to different choices for pre-processing and parameter settings, e.g., ensembles of semantic spaces, wherein the constituent semantic spaces are built with different parameter settings. Semantic space ensembles have been shown often to lead to better predictive performance than the use of any of the constituent semantic spaces on a range of tasks, including medical synonym extraction [83]. Semantic space ensembles also make it possible to combine different types of corpora in an effective manner [37]; in future work, it would be interesting to combine a blog corpus with corpora from other genres, for instance biomedical and clinical corpora but also other corpora in which layman terminology is likely to be used. Finally, a manual curation of the seed term set, before using it for expanding the vocabulary, might be worth exploring, e.g., by removing seed words from existing vocabularies that are atypical for the semantic category in question.

## Conclusions

We have studied different pre-processing strategies and parameter settings for expanding a medical vocabulary with terms belonging to the three semantic categories Medical Finding, Pharmaceutical Drug and Body Part. A scenario was simulated, in which vocabulary lists of 100 terms of each semantic category would be available, and in which a medical terminologist, for instance, would manually scan through a list of candidate terms in search for new terms to add to the vocabulary lists of the investigated semantic categories. The candidate lists were produced by applying random indexing on a Japanese patient blog corpus, and recall against existing Japanese medical vocabularies was measured for a scenario in which the medical terminologist would scan through the top *n* to the top 10*n* terms in the generated candidate lists, where *n* is the number of terms in existing vocabularies of the category in question, i.e., the number of terms that the evaluated system should aim to find.

It could be concluded that different settings for expanding a vocabulary were suitable for different semantic categories. Retaining case particles in the pre-processing of the corpus and using a large context window size was most successful for expanding the list of Body Part terms, while removing case particles and using a small context window was better for the categories Medical Finding and Pharmaceutical Drug. To use proximity to centroids of clusters with a maximum size of 14 seed terms or to the centroid of all 100 seed terms were slightly better ranking strategies when extracting Pharmaceutical Drugs from a term list of 10*n* candidates than to use the centroid of clusters with a maximum size of 90 terms. For the other two investigated categories, however, different cluster sizes only had a very marginal effect on the results for the best pre-processing settings, when looking at recall for a list of the top 10*n* candidates. The most suitable settings, however, also depended on the number of candidate terms that the simulated medical terminologist would be willing to scan through. For instance, when comparing recall for the top *n* candidates for Body Part, proximity to centroids of clusters with a maximum size of two seed terms in each cluster was slightly more successful than using the centroid of larger clusters.

The best pre-processing, context window size and clustering settings resulted in the best average recall values for Pharmaceutical Drug, for which a recall of 25 % was achieved for top *n* candidates and a recall of 68 % for top 10*n*. For a candidate list of top 10*n* candidates, the second best category was Medical Finding, for which a recall of 16 % was achieved for top *n* and a recall of 58 % for top 10*n* candidates. When only taking the top *n* candidates into account, however, results for Body Part were better than for Medical Finding with a recall of 23 %. For the top 10*n* candidates, on the other hand, the strategy was least successful for Body Part, with a recall of 46 %. A medical terminologist would thereby, for instance, be able to expand the hypothetical, small vocabulary with at least 203 new Medical Findings and 119 new Pharmaceutical Drugs by scanning through 1700-2000 candidate terms.

These results demonstrate that the pre-processing and parameter settings applied were successful. They also show the potential in using large corpora for semi-automatic medical vocabulary expansion, not only those comprising formal biomedical texts written in English or similar languages — which have been used in previous studies — but also texts that differ in style and language, such as the Japanese patient blog texts that have been used here.

We hope that the results of this study will inspire an increased use of semi-automatic medical vocabulary expansion methods, for medical vocabularies in a larger range of languages, as well as for vocabularies that are adapted to a wider variety of medical text genres. This, in turn, might widen the types of texts to which important medical text mining applications can be applied; applications such as syndromic surveillance or detection of adverse drug reactions.

## Endnotes

^1^ MeSH: https://www.nlm.nih.gov/research/umls/source releasedocs/2008AB/MSHJPN/mrsab.html MedDRA/J: https://www.nlm.nih.gov/research/umls/sourcereleasedocs/ current/MDRJPN/sourcerepresentation.html.

^2^ The lists of analysed terms (top 100 terms for each of the 500 folds), that were not included in current vocabularies can be found at: http://people.dsv.su.se/~mariask/resources/japanese_vocabulary/.
